# Serial Postoperative Blood and Peritoneal Fluid Analysis and Ultrasonographic Assessment in Client-Owned Horses Undergoing Ventral Midline Exploratory Celiotomy for Colic

**DOI:** 10.3390/ani16172720

**Published:** 2026-09-01

**Authors:** Kayla Kownurko, Margaret Mudge, Laura E. Selmic, Eric Schroeder, Alison Gardner, Laurie Millward, Hilary Rice

**Affiliations:** 1 Department of Veterinary Clinical Sciences, College of Veterinary Medicine, The Ohio State University, Columbus, OH 43210, USA; kayla.kownurko@gmail.com (K.K.); selmic.1@osu.edu (L.E.S.); schroeder.257@osu.edu (E.S.); gardner.300@osu.edu (A.G.); rice.871@osu.edu (H.R.); 2 Department of Veterinary Biosciences, College of Veterinary Medicine, The Ohio State University, Columbus, OH 43210, USA; millward.7@osu.edu

**Keywords:** colic, peritoneal fluid, L-lactate, postoperative complications

## Abstract

Postoperative monitoring, including evaluation of peritoneal fluid, is used to aid in the diagnosis and treatment plan for complications after colic surgery. The primary objective of this study was to determine serial venous and peritoneal postoperative variables, including L-lactate values in horses after ventral midline exploratory celiotomy. A secondary objective was to evaluate postoperative abdominal ultrasonography as a predictor of complications. A total of 36 client-owned horses with signs of colic undergoing ventral midline exploratory celiotomy were prospectively enrolled in the study. Peripheral venous blood and peritoneal fluid were obtained, and abdominal ultrasonographic examination was performed preoperatively (T0) and at 48 h (T1), 72 h (T2), and 120 h (T3) postoperatively. Postoperative ultrasonographic examination suggested a normalization in postoperative small intestinal diameter, both in horses that experienced complications and those that did not. Peripheral L-lactate at T0, T1, T2, and T3 was 1.9 mmol/L (±1.5), 0.9 mmol/L (±0.5), 0.8 mmol/L (±0.3), and 0.9 mmol/L (±0.3), respectively. Peritoneal L-lactate at T0, T1, and T2 was 3.8 mmol/L (±2.4), 2.8 mmol/L (±1.8), and 1.1 mmol/L (±0.9). T3 was unable to be evaluated due to insufficient fluid. Of the 32 horses that survived to discharge, 25/32 experienced postoperative complications. The most common complication was postoperative colic. Postoperative peritoneal L-lactate was higher than blood L-lactate, even in horses without postoperative complications. Postoperative blood L-lactate was higher across all timepoints in horses with complications. This study was limited by the small number of cases.

## Simple Summary

Colic is a common equine emergency condition, and ventral exploratory midline celiotomy (colic surgery) is commonly performed in horses that present to tertiary emergency referral hospitals for colic. Postoperative challenges after ventral midline exploratory celiotomy include persistent pain, postoperative reflux, anastomotic leakage or obstruction, peritonitis, and non-viable (dead) intestine. When these complications arise, peritoneal fluid is often obtained to gather more information about whether a repeat celiotomy is needed. Although peritoneal fluid analysis, including L-lactate, is commonly used to detect compromised bowel preoperatively, there is minimal information to guide interpretation of peritoneal fluid in the immediate postoperative period for horses after colic surgery. Our objectives were to determine common peritoneal and blood postoperative variables in horses preoperatively and 48, 72, and 120 h after colic surgery. Additionally, we obtained transabdominal ultrasonographic measurements at the same timepoints. Peritoneal L-lactate and small intestinal diameter decreased over time. Peritoneal fluid color remained abnormal, and peritoneal L-lactate was higher than blood L-lactate, regardless of the outcome or complication status. Venous blood L-lactate was higher across all timepoints in horses with postoperative complications.

## 1. Introduction

Complications after colic surgery are common, and while many complications are self-limiting or medically manageable, severe complications such as anastomotic obstruction, intestinal leakage, postoperative reflux, and intra-abdominal adhesions can result in repeat celiotomy or euthanasia [1]. Many of the diagnostic clinical procedures used for initial colic workup can also be used to investigate colic and other clinical complications in the postoperative period. Clinical variables (e.g., physical examination, pain assessment, nasogastric reflux, and abdominal ultrasonography) are commonly used to determine the need for repeat celiotomy [2]. Jacobs et al. (2019) investigated horses with postoperative reflux after small intestinal surgery, and found that of the horses requiring repeat celiotomy, the median time to repeat celiotomy was 5 days [3]. The authors concluded that horses that developed fever and colic in the postoperative period after small intestinal surgery were more likely to have a surgical reason for the presence of postoperative reflux. Postoperative blood and peritoneal fluid postoperative variables and abdominal ultrasonographic findings were not reported in this study, but could potentially be useful for earlier or more accurate detection of a surgical lesion.

There is limited veterinary literature describing postoperative peritoneal fluid and abdominal ultrasonography of the horse after ventral midline celiotomy. A study by Epstein et al. (2008) investigated postoperative ultrasonographic findings in ponies after bowel handling at days 1, 3, 5, and 7 postoperatively [4]. The findings were suggestive that there was an inflammatory component to bowel handling, which was reflected in increased small intestinal mural thickness and motility that normalized over the study period [4]. Another study, by Cuevas-Ramos et al. (2019), examined postoperative ultrasonographic images and videos in horses after ventral midline exploratory celiotomy [5]. Horses that had large colon lesions exhibited similar small intestinal motility to the control horses, and those that had small intestinal lesions were more likely to have changes related to their small intestine mural thickness, diameter, and motility postoperatively.

Point-of-care testing is routinely used to evaluate peritoneal fluid in horses during colic workup, and the comparison of peritoneal to peripheral L-lactate is well established as a determinant of intestinal ischemia and the need for emergency celiotomy [6,7,8]. Total nucleated cell counts (TNCCs) and total protein (TP) in peritoneal fluid have been shown to increase dramatically after experimental ventral midline celiotomy, experimental small colon resection and anastomosis, and experimental jejunocecostomy in horses [9,10,11]. However, information about postoperative peritoneal fluid values in clinical cases is limited [12]. Even in the absence of gastrointestinal disease or manipulation, the TNCC and TP have been shown to markedly increase after elective laparoscopic cryptorchidectomy and ovariectomy in horses [13,14].

This study was completed prior to the publication of a recent study by Granello et al. that provides the most information, to date, about postoperative peritoneal fluid analysis in horses with naturally occurring gastrointestinal lesions [12]. In the Granello study, blood lactate and peritoneal lactate were measured in preoperative or intraoperative colic cases and at 24 h, 48 h, and 168 h postoperatively using a lactometer that has been previously validated for use on equine blood and peritoneal fluid (Lactate Plus) [15]. Due to the paucity of literature evaluating peritoneal markers of ischemia and inflammation in the postoperative period prior to this publication, there is a need for further research about the values for point-of-care testing and fluid cytology in naturally occurring postoperative colic cases. The current “gold standard” is TNCC, TP, cell differential, and cytology of peritoneal fluid, but interpretation is complicated by the robust inflammatory response in the immediate postoperative period [16]. While the decision to perform a repeat celiotomy or pursue more aggressive medical care is commonly multifactorial, more information to guide interpretation of postoperative peritoneal fluid would allow clinicians to be more confident in their recommendations to owners postoperatively. Prompt recognition of horses requiring a repeat celiotomy is important because a delay in surgery will likely worsen the prognosis. On the other hand, additional surgery carries more cost to owners, as well as anesthetic and recovery risk to the horse and higher incidence of incisional infection [17].

Our aim was to perform an exploratory study of serial point-of-care testing to evaluate blood and peritoneal fluid postoperative variables in horses after colic surgery. Based on the available literature regarding timing of common postoperative gastrointestinal complications, sampling was performed at 48, 72, and 120 h postoperatively, and a lactometer validated for horse blood and peritoneal fluid was utilized (the same as that in the Granello study) [15]. A secondary objective was to evaluate postoperative abdominal ultrasonographic findings and blood and peritoneal fluid as predictors of complications. We hypothesized that blood and peritoneal L-lactate values would serially decrease over time after surgery, and horses with complications would have higher peripheral and peritoneal L-lactate values. The secondary hypothesis was that horses with no major complications would have improved small intestinal motility and decreased small intestinal diameter over time.

## 2. Materials and Methods

All procedures were approved by the Institutional Animal Care and Use Committee (protocol #2024A00000019) and reviewed and approved by the Privately Owned Animal Subcommittee at The Ohio State University. Client consent was obtained for all procedures prior to initiation of the study protocol.

### 2.1. Animals and Experimental Design

The study design was a prospective observational descriptive cohort study. Client-owned horses undergoing ventral midline celiotomy due to acute abdominal pain that recovered from general anesthesia were prospectively enrolled. Horses were excluded if intestinal disease was not identified at surgery, or if the horse was <1 year old. At the time of the study design, no comparable data regarding serial postoperative peripheral and peritoneal L-lactate values were available to generate a sample size that would detect a difference between timepoints. The TNCCs from Santschi et al. (1988) at the 72 h and 120 h timepoints were used as a related variable to provide an estimate of the expected effect of ventral midline celiotomy on peritoneal fluid [10]. The TNCC of 127,750 cells/µL +/− 46.252 at 72 h compared to a mean of 81,333 cells/µL at 120 h was used as a reference. Based on the clinical cases at our hospital, an effect size of 1 mmol/L with a standard deviation of 0.8 mmol/L for L-lactate was proposed, yielding a minimum sample size of 24 horses. Enrollment was extended to 36 horses with IACUC approval due to the inability to obtain all timepoints for most horses, as well as the apparent low risk to the horses.

### 2.2. Sample Collection and Analysis

Venous blood and peritoneal sampling and point of care testing were performed preoperatively (T0) and at 48 h (T1), 72 h (T2), and 120 h (T3) postoperatively. Point of care testing was performed as described for venous blood. Venous blood was sampled (10 mL) by direct jugular venipuncture, and blood was collected into EDTA and serum tubes (BD vacutainer, Franklin Lakes, NJ, USA). Abdominocentesis was attempted at all timepoints unless there was a contraindication (behavior concerns, distended bowel, or enlarged spleen). The horse was sedated with xylazine (0.3–0.5 mg/kg IV) or detomidine (0.006–0.02 mg/kg IV) +/− butorphanol (0.006–0.02 mg/kg IV) and restrained using a nose twitch (nasolabial restraint). Abdominocentesis was performed using either an 18G 1.5″ or 3″ needle or teat cannula using ultrasonographic guidance (either real-time or body wall measurement) after sedation, skin preparation, and local anesthesia with 2% lidocaine. As much peritoneal fluid as possible was obtained. Location, fluid character, volume obtained, fluid characteristics (i.e., color, turbidity), and image measurement (depth to fluid) were recorded. Peritoneal fluid was collected into EDTA and serum tubes (BD vacutainer, Franklin Lakes, NJ, USA).

L-lactate, packed cell volume, total protein, and glucose were measured immediately after sample collection (venous whole blood and peritoneal fluid) with point-of-care analyzers (Nova Biomedical Lactate Plus, Waltham, MA, USA; AlphaTrak3, Zoetis, Kalamazoo, MI, USA). Serum amyloid A (SAA) was processed to serum and measured on serum samples using the Stablelab^®^ Lab version test kit per manufacturer instructions (Zoetis, Kalamazoo, MI, USA).

Cytology, TNCC, and TP were performed by the OSU Clinical Pathology Service when there was enough fluid obtained (>0.5 mL). Total nucleated cell count was determined by processing the sample on the ADVIA 2120 (Siemens Healthcare Diagnostics Inc., Tarrytown, NY, USA), and TP was measured using refractometry.

### 2.3. Point-of-Care Ultrasonographic Protocol

A GE Logiq E NextGen ultrasonographic machine (GE Medical Systems, Wuxi, China) was utilized with a convex transducer (1.5–5 MHz). Measurements were made with the electronic calipers associated with the ultrasonographic software [18]. A modified FLASH (Fast Localized Abdominal Sonography of Horses) protocol was performed at each postoperative timepoint (T1, T2, and T3) as established by Busoni et al. 2011, with the modifications being the cranioventral abdomen, which was used as the 7th window on the right instead of the cranioventral thorax, and an inguinal window was added in the right and left inguinal regions [19]. Video clips (3–5 s) were obtained at both the right and left inguinal regions. Still images were obtained at each of the established FLASH sites and the intended abdominocentesis site (modified 7th window) at clinician/resident discretion. Information recorded included maximum jejunal diameter, motility (absent, decreased, normal, and increased), and mural thickness in the inguinal regions. Large colon mural thickness, caudal extent of the stomach, and peritoneal fluid were also recorded. The loops and images were evaluated by a blinded, double-boarded large animal surgeon/criticalist to categorize small intestinal motility and peritoneal effusion categorization. These variables were modifications of the Epstein et al. protocol [4,20]. Mural thickness of the jejunum was measured from a hyperechoic line representing the serosa to the hyperechoic line representing the mucosal surface, and if more than one measurement was made, the mean was used. Thickened jejunal wall was defined as >3 mm. Jejunal contractility was assessed qualitatively: absent = no waves; hypocontractile = infrequent; weak peristaltic waves, normal contractility = frequent; and rhythmic peristaltic waves, or hypercontractile = constant peristaltic waves with extended periods of contraction. At the right cranioventral abdomen, peritoneal fluid was subjectively quantified as none, transiently visible (fluid disappeared as intestinal contents shifted and viscera contracted and expanded while being imaged), small amount (fluid was present during the entire time the region was imaged and when the probe was returned to the region after examining another region and <2 cm), or large amount (surrounding viscera and ≥2 cm). The peritoneal fluid was also characterized as anechoic or anechoic with hyperechoic flecks.

### 2.4. Clinical Data Collection and Classification

Clinical information recorded included signalment, diagnosis, and surgical procedures (enterotomy and intestinal resection), postoperative complications, and survival to discharge. Heart rates were also recorded at each timepoint. Postoperative complications were categorized as fever >38.9 °C (>102 °F), colic (clinical signs of colic requiring additional analgesia and/or passage of a nasogastric tube), surgical site/incisional infection (surgical abdominal wound draining over 24 h after colic surgery), septic peritonitis (fever and positive bacterial culture for Gram-negative species and peripheral to peritoneal glucose difference of 50 mg/dL), diarrhea (increased fecal water content > 3 piles within 24 h), and reflux (reflux volume > 20 L during a 24 h period after surgery or a volume > 8 L on one occasion after surgery) [21,22].

The complications were then categorized using a modified accordion severity classification system as either no complications (score of 0), mild complications (score of 1), moderate complications (score of 2), severe complications (score of 3), and death (score of 4) [23,24]. Mild complications were categorized as a transient fever (resolved in less than 12 h without medical intervention other than routine nonsteroidal anti-inflammatory medication) and/or mild self-limiting colic. Moderate complications were categorized as colic requiring medical treatment (e.g., pain medication, IV fluids, and/or passage of nasogastric tube), reflux, *Salmonella* shedding in feces, and surgical site infection. Severe complications were categorized as repeat celiotomy, ongoing colic signs, or complications with long-term effects or disability, including euthanasia based on complications, but not deemed to have a poor to grave prognosis. Death was defined as natural death or euthanasia due to poor or grave prognosis. Additionally, specific gastrointestinal complications were defined as colic, nasogastric reflux, and diarrhea.

### 2.5. Statistical Analysis

All statistical analyses were performed using SAS software, version 9.4 (SAS Institute Inc., Cary, NC, USA). Continuous variables were assessed for normal distribution using summary statistics and graphical inspection and are reported as mean ± standard deviation or median (range), as appropriate. Categorical variables are reported as counts and percentages. Observations with missing outcome or covariate data were excluded on a per-analysis basis.

Binary outcomes, including the presence of postoperative complications, infectious versus non-infectious complications, strangulating lesions, septic peritonitis, and survival to discharge, were evaluated using logistic regression models. Univariable and multivariable models were constructed with predictors selected a priori based on biological plausibility and clinical relevance, including age, lesion location (small intestine vs. large intestine), strangulating status, enterotomy, intestinal resection, venous lactate, peritoneal lactate, the peritoneal-to-venous lactate ratio, lactate delta, serum amyloid A (SAA), gastrointestinal-specific complications, and an ordinal postoperative complication score. Model discrimination was assessed using the area under the receiver operating characteristic curve (AUC). In instances of sparse data or complete separation, model convergence diagnostics were evaluated, and results were interpreted descriptively.

To examine survival outcomes, logistic regression models were used with survival to hospital discharge as the binary outcome. Separate survival models were constructed to evaluate the effects of preoperative clinical variables and perioperative factors, as well as postoperative complication score and the presence of gastrointestinal-specific complications.

For longitudinal postoperative analyses, generalized linear mixed-effects models were used to evaluate repeated binary outcomes (e.g., occurrence of complications, performance of abdominocentesis) using a logit link function. Horse identification was included as a random effect to account for repeated observations within individuals. Models were fit using residual pseudo-likelihood estimation with containment method degrees of freedom. Model convergence was assessed using optimization diagnostics.

Repeated continuous variables, including venous lactate, peritoneal lactate, and serum amyloid A concentration, were analyzed using linear mixed-effects models. Fixed effects included timepoint, complication status, and their interaction, with horse identification included as a random effect to account for within-horse correlation. Models were fit using restricted maximum likelihood estimation with a variance components covariance structure. Type III tests of fixed effects were used for inference.

All statistical tests were two-sided, and significance was set at *p* < 0.05.

## 3. Results

### 3.1. Demographics and Surgical Details

A total of 36 horses were enrolled in the study from July 2024 to November 2025. Mean age was 12 years (+/−7.1; range: 2–28), with 14 mares, 20 geldings, two stallions, and a variety of breeds (nine Quarter Horses, five Warmbloods, four Thoroughbreds, three Morgans, three ponies, two American Paint Horses, two National Show Horses, two Tennessee Walking Horses, two Standardbreds, two drafts, one Mustang, and one Lusitano). The mean heart rate at the time of presentation was 58 bpm (+/−19.3; range 32–120). Eighteen horses had small intestinal lesions (50%) and 18 had large intestinal lesions (50%). Of the horses with strangulating lesions, 12 had strangulated small intestine that required resection and anastomosis as follows: nine jejunojejunostomies, two jejunocecostomies, and one jejunoileocecostomy. One horse with a strangulating small colon lesion required resection and anastomosis. Two strangulating lesions were large colon (no resection performed). Enterotomy was performed in 28 horses (78%; 14 jejunal, 28 large colon, three small colon, and two cecal). Intestinal resection was performed in 17 horses (47% of horses). Of the resections, 13 were strangulating lesions (see above) and three were small intestinal obstructive lesions. One cecal resection was performed. Heart rate was not a significant predictor of complications at any timepoint.

### 3.2. Blood and Peritoneal L-Lactate

Venous L-lactate was measured at the time of admission for all horses. Peritoneal L-lactate was measured in 30/36 horses. The results are reported in Table 1. Venous L-lactate was significantly higher in horses with any postoperative complication (*p* = 0.0435), with no significant time interaction. In horses with no postoperative complications, venous L-lactate at T0, T1, T2, and T3 was 1.3 ± 0.4 mmol/L, 0.7 ± 0.1 mmol/L, 0.6 ± 0.2 mmol/L, and 0.8 ± 0.3 mmol/L, respectively. In horses with any postoperative complications, venous L-lactate at T0, T1, T2, and T3 was 2.1 ± 1.7 mmol/L, 0.9 ± 0.6 mmol/L, 0.8 ± 0.3 mmol/L, and 1.0 ± 0.3 mmol/L, respectively.

Multivariable logistic regression using lactate ratio, lactate delta, age, and small intestinal abnormalities showed excellent preoperative discrimination for strangulation (AUC 0.95). A total of 22 postoperative peritoneal fluid samples were available for analysis. Of these 22, one sample was eliminated from analysis after it was determined to be an enterocentesis sample. A total of 16/36 samples had peritoneal fluid that was able to be obtained at either 48 h (12/36), 72 h (8/36), or 120 h (2/36) (Table 2). There were not enough peritoneal samples obtained at T3 for analysis due to a combination of difficulty obtaining fluid as well as the decision by the investigator to limit repeated attempts in cases of patient non-compliance, severe ventral edema, and extensive large intestinal gas or fluid distension. Only one horse was able to obtain sufficient abdominal fluid for analysis at all three timepoints. This was due to both unsuccessful abdominocentesis attempts, euthanasia prior to the last timepoint in four horses, and discharge from the hospital prior to the last timepoint in four horses. No postoperative peritoneal lactate variables were significantly associated with strangulating lesions or with postoperative complications.

There was no association between peripheral to peritoneal lactate difference or ratio and postoperative complications. There were six horses from which peritoneal fluid was not collected preoperatively. Of these six horses, abdominocentesis attempts were made postoperatively, but five of these horses had no peritoneal fluid collected at any timepoint. Of the horses from which peritoneal fluid was able to be obtained at the 48 h timepoint (12), 11/12 horses experienced complications (four had mild complications, three had moderate complications, and four had severe complications, or death). Of the horses from which fluid was able to be obtained at the 72 h timepoint, 8/8 experienced complications, with 3/8 experiencing mild complications, 3/8 experiencing moderate complications, and 2/8 experiencing severe complications or death. Of the horses from which peritoneal fluid was able to be obtained at any timepoint postoperatively, 12/16 survived to discharge. In the four horses who did not survive to discharge, fluid was able to be obtained at all attempted timepoints prior to euthanasia. Of the 14 horses from which peritoneal fluid could not be obtained, 6/14 did not experience complications and 8/14 did. Of the eight horses who experienced postoperative complications, all complications were mild.

### 3.3. Additional Blood and Peritoneal Postoperative Variables

Glucose and total protein are reported for blood and peritoneal fluid in Table 1 and Table 2, respectively. PCV, SAA, and total WBC concentration are reported for venous blood in Table 1. Total nucleated cell count is reported for peritoneal fluid in Table 2.

There was no association between blood glucose, SAA, and total WBC count with postoperative complications. SAA alone did not differentiate infectious from non-infectious complications. WBC was not routinely measured in preoperative peritoneal samples, and there were missing data points for some horses at other timepoints due to insufficient sample volume.

All peritoneal fluid samples obtained at T1, T2, and T3 were serosanguineous, regardless of postoperative complications. Peritoneal lactate, peritoneal:blood lactate ratio, and peritoneal–blood lactate difference are reported in Table 2. Peritoneal glucose was lower than venous blood glucose in all but two samples. The mean difference between blood and peritoneal glucose at both T1 and T2 was 26 mg/dL. There were only three samples that had a difference of >50 mg/dL. One of these cases had confirmed peritonitis (blood–peritoneal lactate difference of 88 mg/dL). The other two horses had mild, transient, postoperative fevers, but no specific GI complications. There was sufficient peritoneal fluid for TNCC in 15 horses and 22 samples (Table 2). There were only two samples obtained at T3. One horse had septic peritonitis (TNCC 258,030/µL), and one horse had severe postoperative reflux (56,790/µL). TNCC was not included in the logistic regression model due to the low number of samples.

### 3.4. Postoperative Ultrasonography

The small intestinal contractility distribution was available as a complete data set in 33/36 horses (Supplementary Table S1). There were no instances of hypermotile small intestine identified at any timepoint. Small intestinal diameter decreased over time (T0 mean 4.5 +/− 1.8 cm, T1 mean 3.8 +/− 1.4 cm, T2 mean 3.7 +/− 1.3 cm, and T3 mean 2.7 +/− 0.6 cm). The small intestinal diameter and small intestinal mural thickness results are available in Table 3. The small intestinal diameter normalized in the postoperative period, both in horses that experienced complications and those that did not.

The volume of peritoneal fluid was not statistically associated with complications. A total of 23 horses had no free fluid at any postoperative timepoints. Five horses had a transient amount at at least one timepoint, six had a small amount at at least one timepoint, and two had a large amount at at least one timepoint. Of the eight cases with a small or large amount of peritoneal fluid, three of these cases were subject to euthanasia and two were discharged from the hospital. However, these two horses experienced severe complications. Of the horses that were discharged, one horse was diagnosed with septic peritonitis, where cytology was able to aid in the diagnosis and management, and one horse that had jejunocecostomy experienced severe postoperative colic and postoperative reflux and distended small intestine. Repeat celiotomy was recommended at 48 h postoperatively due to concern for obstruction of the anastomosis site. The owner declined surgery and the horse was discharged from the hospital after intensive medical management of persistent abdominal pain and postoperative reflux. Of the three that were not discharged, all three experienced severe complications and were subject to euthanasia. Two of these horses experienced colic that was non-responsive to medical management, and repeat celiotomy was not an option. One horse had persistent postoperative reflux; repeat celiotomy was performed, no anastomotic issues were found, and the postoperative reflux did not resolve.

### 3.5. Postoperative Complications

Thirty-two horses were discharged from the hospital (89%). Of the four that did not survive to discharge, two underwent repeat exploratory celiotomy. Of those two, one horse sustained a catastrophic fracture in recovery, and the other recovered and was subject to euthanasia due to continuing postoperative reflux. Of the two that did not have a repeat celiotomy and were subject to euthanasia, one had intractable signs of colic and did not have a financial option for additional surgery, and the other collapsed and was unable to rise (definitive diagnosis not obtained). The distribution and severity of complications across different intestinal lesion types is available in Table 4.

There were three complications related to postoperative abdominocentesis: one splenocentesis, one enterocentesis, and one focal cellulitis at the abdominocentesis site. These horses were discharged from the hospital without additional complications. Of the 32 horses that survived to discharge, eight had no complications, 15 had mild complications, seven had moderate complications, and three had severe complications. Postoperative colic was the most common complication in 10/32 horses (31%), followed by reflux in 9/32 horses (28%), surgical site infection in 3/32 horses (9%), and septic peritonitis in 1/32 horses (3%). The one horse with septic peritonitis responded to medical management and was discharged from the hospital.

Complications could not be predicted based on the preoperative variables of venous lactate, age, and operative details of small intestinal versus large intestinal lesions, strangulating versus non-strangulating lesions, enterotomy, or resection. Similarly, these variables could not predict survival to discharge. Regarding survival, any GI complications increased the risk of non-survival, with multiple GI complications trending toward worse survival.

## 4. Discussion

This study provides additional information about postoperative variables in clinical colic cases. The commonly used variables of peritoneal fluid color and lactate do not appear to be reliable predictors of complications in horses after ventral midline celiotomy.

The primary objective of this study was to determine venous and peritoneal postoperative variables in ventral midline exploratory celiotomy cases at 48, 72, and 120 h post operatively. Our data showed that postoperative peritoneal lactate was higher than venous lactate at all timepoints (48 h, 72 h, and 120 h postoperatively) in horses with and without complications. In horses with complications, venous lactate was significantly higher across all timepoints than in horses without complications. Preoperative venous L-lactate appears to be predictive of complications [25,26]. Postoperative venous L-lactate appears to be statistically predictive of complications. However, the mean values in horses with complications, while higher than those without complications, were still within normal limits (<2 mmol/L). Therefore, biologic relevance is questionable, and given the small sample size these findings should be interpreted cautiously.

We found that at all timepoints postoperatively, in horses with and without complications, the peritoneal fluid obtained was serosanguineous. This suggests that serosanguineous peritoneal fluid at any timepoint postoperatively is not consistent with diagnosis of ischemic bowel, and must not be used clinically as a sole index for ischemic bowel and repeat exploratory celiotomy. Both the serosanguineous peritoneal fluid postoperatively and the peritoneal fluid lactate being higher than the peripheral lactate postoperatively is consistent with the results published by Granello et al. [12]. The venous lactate being significantly higher in any horses experiencing postoperative complications at any timepoint postoperatively is consistent with previously reported serial venous lactate measurement in horses after colic surgery [27]. Our findings also reinforced that preoperative peritoneal to peripheral lactate difference is the most suggestive of strangulating lesions at surgery, although this did not reach statistical significance, likely due to low case numbers and varied surgical lesions.

It is known that peritoneal fluid is an ultrafiltrate of plasma, so in the absence of gastrointestinal ischemia or infection, peritoneal lactate should be similar or lower than blood or plasma lactate [6]. One possible explanation for peritoneal fluid lactate remaining higher than the peripheral lactate is that the underlying gastrointestinal lesion may have a greater impact on peritoneal fluid compared to venous blood. The disease process that results in the increase in peritoneal lactate in strangulating lesions and the switch to anaerobic metabolism (yielding lactate) may take longer to clear from peritoneal fluid compared to blood or plasma. Trauma sustained to the bowel and mesentery during surgical manipulation may also result in inflammation and altered glucose metabolism, potentially contributing to persistently increased peritoneal lactate. Clearance of lactate from the peripheral blood should occur quickly, both due to correction of the lesion and appropriate fluid support allowing for metabolism of L-lactate via the Cori cycle within the liver through gluconeogenesis. Additionally, although the peritoneal lactate was higher than venous blood lactate in all horses, the horse with septic peritonitis had a much higher peritoneal: blood lactate ratio and difference as well as a very low peritoneal fluid glucose. Based on this single instance of confirmed septic peritonitis, fluid postoperative variables and cytology should be diagnostic. Additionally, perhaps with a larger sample pool of horses, cut-off values for postoperative intestinal compromise or septic peritonitis could be developed. Another potential explanation for a higher postoperative peritoneal fluid lactate is neutrophil metabolism. Because the TNCC in the postoperative time periods was higher than the established reference range, intraperitoneal neutrophil metabolism, which is primarily dependent on glycolysis, could result in a higher postoperative peritoneal lactate relative to venous blood [28,29].

Obtaining peritoneal fluid postoperatively was difficult. Because these were clinical cases and not experimental cases, abdominocentesis attempts were limited. As many of the abdominocentesis attempts were not triggered by a clinical need, investigators were more likely to terminate repeated abdominocentesis attempts than if peritoneal fluid would have been useful in clinical decision-making. This clinical judgment could have skewed the data, in that the horses for which fluid was most consistently obtained may have been those that were experiencing complications and producing increased peritoneal fluid. In addition, horses at the third timepoint may have been more likely to have ventral edema and more ingesta in the colon. This may explain the low sample size at T3. In general, the likelihood of obtaining fluid decreased substantially after the 48 h timepoint. There are a few possible explanations for this. Peritoneal fluid may have been reabsorbed via lymphatics postoperatively, making it challenging to obtain fluid. Further, there is some evidence to support that in an inflammatory state, there is an increase in resorption of fluid from the peritoneal cavity, potentially limiting the fluid available for sampling [30]. It is also possible that peritoneal fluid leaked through the defect in the parietal peritoneum into the retroperitoneal space, since that layer was not directly closed at the time of surgery. There is also a possibility that it was more difficult to penetrate the peritoneum during abdominocentesis since the ventral parietal peritoneum was not fully intact, or the parietal peritoneum may have been thicker (due to inflammation), and therefore more difficult to penetrate. There were times when at the discretion of the investigator performing the ultrasonographic examination on the horse, fluid was not attempted to be obtained, or the attempt was limited (i.e., one attempt, no additional attempts) due to lack of compliance of the horse, extensive large intestinal gas or fluid distention, or excessive ventral edema. Granello et al. appeared to be more successful in obtaining fluid postoperatively, possibly due to the decision to set their first timepoint at 24 h [12]. According to Epstein et al., peritoneal fluid is most likely to be seen on ultrasonographic examination at the 24 h timepoint [4]. The investigators may also have been more persistent in attempts to obtain peritoneal fluid in their cases. Another difference may be routine surgical procedures, in that we routinely perform saline lavage at the end of surgery and suction out all remaining fluid prior to abdominal closure.

A subset of horses (eight) had either a small or large amount of free peritoneal fluid on postoperative ultrasonographic examination, and 4/8 horses fell into the severe complication/death group. This could suggest that detection of free peritoneal fluid postoperatively may be associated with complications.

In the recently published study of serial peritoneal fluid analysis, Granello et al. reported significantly higher peritoneal total protein preoperatively in horses with strangulating lesions, and the total protein remained high in all horses postoperatively [12]. Our data did not appear to identify higher peritoneal total protein in strangulating cases. Postoperative peritoneal protein values remained high at 48 and 72 h in all lesions (not enough data at the T3 timepoint).

Postoperative peritoneal fluid TNCC was greater than the accepted reference range of less than 5000 cells/µL at all timepoints. The mean TNCC at T1 and T2 in our study (approximately 19,000 cells/µL and 15,000 cells/µL, respectively) is far lower than the TNCC previously reported by Hanson et al. 24 h postoperatively (approximately 130,000 cells/µL and 167,000 cells/µL for non-resection and small colon resection in research horses) and Santschi et al. (a mean of 138,000 cells/uL) [10,11]. In these previous experimental studies, there was a decrease in TNCC by day 6–7 (40,000–43,000 cells/µL). An explanation for why our T1 and T2 may have been lower than that reported by Santschi could be that our horses were abdominally lavaged with saline immediately prior to closure, which could have caused a decrease in postoperative inflammation. The horses in the Hanson study were lavaged with LRS and heparin, but those horses did a have a drain placed, which may have negated the anti-inflammatory effects of the lavage. An additional consideration could be the time delay (48 h for our study versus 24 h for the Santschi study). However, at comparable times (i.e., 3 days postoperatively), the TNCCs from the Santschi and Hanson studies are still well above our mean TNCC at 2 days postoperatively.

Similarly, of the eight horses that had TNCCs available in the Granello study at the 24 h and 168 h timepoints, all eight samples reflected an overall downward trend in TNCC [12]. Our first two timepoints showed a decrease in TNCC, but the third timepoint was markedly increased. This is likely due to the fact that these horses had marked defined complications (one septic peritonitis and one postoperative reflux), and thus these values do not reflect the trend toward normalization seen in other studies.

Regarding postoperative variables, SAA was not predictive in our study of identifying complications, which is in contrast with previous studies that indicate that SAA is an early indicator of complications [31,32]. This difference may be related to systemic stabilization of our cases or low sample size. Sampling timepoints may also have prevented detection of a significant difference, since SAA was not measured daily. Our timepoints were selected based on when complications and decision-making regarding repeat celiotomy were most likely to occur. We also did not find a significant association between admission heart rate or PCV with outcome or complications. These variables have been associated with outcome in previous studies [33,34] Our case population was variable and did not include a large number of large colon volvulus cases, which may have limited the predictive value of heart rate and PCV. This also may have been in general due to not having enough cases to detect a difference or that we did not analyze our data for this value on the basis of lesion, which may have resulted in significance if divided into, for example, strangulating small intestinal lesions versus large colon displacements (no ischemia).

Postoperative abdominal ultrasonography is often used to investigate postoperative colic and persistent reflux, but findings can be difficult to interpret because there is not extensive information about typical postoperative abdominal ultrasonographic examination findings. Cuevas-Ramos et al. evaluated postoperative ultrasonographic images and videos in horses with small intestinal lesions with and without anastomosis and concluded that horses with small intestinal lesions had abnormal ultrasonographic findings (decreased small intestinal motility and increased diameter) immediately postoperatively (days 1–3) [5]. In our study, horses had abnormal small intestinal motility and diameter/mural thickness in the immediate postoperative period, but these values normalized over the 120 h of the study. Similarly, our findings reflected those observed by Epstein et al., that peritoneal fluid was most likely to be identified on ultrasonography early in the postoperative time course (at day 1 in their study), typically decreasing thereafter [4].

This study does have a few limitations. The overall case numbers were low and there were a variety of surgical lesions, making it difficult to parse out if there was a correlation between any particular data set for a particular lesion. Additionally, fluid was not able to be obtained at every timepoint. Another potential limitation is the subjectivity of ultrasonographic interpretation. Some subjectivity was unavoidable despite the use of a rubric to determine thickness and motility. And finally, there were only a handful of cases with severe complications, making our data more helpful for describing the typical postoperative peritoneal fluid and on ultrasonographic examination, but not large enough to isolate those cases with complications and to make a robust comparison between the two groups yielding statistical significance.

Some future directions would be to continue to obtain postoperative data in a large number of cases, potentially enabling comparisons between subsets of groups. There are additional postoperative variables that have shown promise, both for venous blood and peritoneal fluid analysis in colic cases. Proteomics, procalcitonin, creatine kinase, cytokines, and neutrophil percentages may provide more specific or sensitive postoperative variables for postoperative intestinal leakage and intestinal compromise [35,36,37,38,39,40].

It is important to emphasize that in horses having complications after colic surgery, the entire clinical picture, including physical exam, bloodwork, ultrasonographic examination, abdominocentesis, and level of comfort are all used to make the clinical decision of whether or not to recommend repeat celiotomy or euthanasia. The most significant driver in the decision to recommend repeat celiotomy in a case is a combination of the clinical picture and degree of pain.

## 5. Conclusions

Serial postoperative blood lactate values alone as well as ultrasonographic examination, including larger peritoneal fluid volume, might be indicative of postoperative complications, with all horses in the high complication group having significantly higher peripheral lactate and postoperative ultrasonographic abnormalities at all postoperative timepoints. Additionally, all timepoints, regardless of complications, yielded serosanguineous abdominal fluid, indicating that postoperative serosanguineous fluid cannot be used as an indicator for repeat celiotomy, as in the postoperative period it is non-suggestive of a strangulating lesion.

## Figures and Tables

**Table 1:** Mean (+/− standard deviation) of venous postoperative variables collected preoperatively and selected times postoperatively in horses undergoing exploratory celiotomy for colic.

Postoperative Variable	Subclassification		T0 (Admission—0 h)	T1 (48 h)	T2 (72 h)	T3 (120 h)
Packed Cell Volume (%)	Strangulating	Large intestinal	32 (6)	29 (5)	31 (2)	32 (2)
		Small intestinal	39 (8)	30 (6)	32 (6)	33 (3)
		Combined	38 (8)	30 (6)	31 (5)	32 (3)
	Non-strangulating	Large intestinal	38 (6)	32 (5)	33 (6)	32 (10)
		Small intestinal	39 (6)	32 (6)	30 (4)	37 (4)
		Combined	38 (5)	32 (5)	33 (6)	33 (9)
Total Protein (g/dL)	Strangulating	Large intestinal	6.2 (0.1)	6.2 (0.7)	6.2 (0.5)	6.2 (0.4)
		Small intestinal	6.6 (0.8)	6 (0.5)	6.4 (0.7)	6.2 (0.8)
		Combined	6.5 (0.7)	6 (0.5)	6.4 (0.7)	6.2 (0.7)
	Non-strangulating	Large intestinal	6.5 (0.7)	6.5 (0.6)	6.4 (1.5)	6.7 (0.3)
		Small intestinal	6.6 (0.9)	6.5 (0.7)	7 (0.6)	6.7 (0.6)
		Combined	7 (1)	6 (1)	6 (1)	7 (0)
Lactate (mmol/L)	Strangulating	Large intestinal	1.5 (0.4)	0.6 (0.2)	0.7 (0.3)	0.9 (0.4)
		Small intestinal	2.5 (2)	1 (0.7)	0.7 (0.3)	0.9 (0.3)
		Combined	2.2 (1.9)	0.9 (0.6)	0.7 (0.3)	0.9 (0.3)
	Non-strangulating	Large intestinal	1.5 (0.9)	0.7 (0.3)	0.9 (0.3)	1 (0.4)
		Small intestinal	2 (1.3)	1 (0.6)	0.6 (.1)	0.9 (0.4)
		Combined	1.6 (1)	0.8 (0.4)	0.8 (0.3)	1 (0.4)
Glucose (mg/dL)	Strangulating	Large intestinal		111 (10)	116 (5)	113 (3)
		Small intestinal		134 (30)	145 (39)	131 (28)
		Combined		128 (29)	139 (36)	127 (26)
	Non-strangulating	Large intestinal		112 (22)	116 (23)	109 (12)
		Small intestinal		132 (28)	125 (38)	128 (23)
		Combined		117 (24)	118 (26)	113 (16)
Serum Amyloid A (mg/L)	Strangulating	Large intestinal		1738 (875)	1781 (854)	848 (183)
		Small intestinal		1384 (709)	1541 (1007)	1395 (836)
		Combined		1473 (728)	1601 (939)	1269 (767)
	Non-strangulating	Large intestinal		1059 (293)	1351 (687)	919 (510)
		Small intestinal		1316 (523)	1322 (404)	
		Combined		1136 (366)	1343 (366)	978 (461)
White Blood Cell Count (×10^9^/L)	Strangulating	Large intestinal		3.2 (3.3)	2.1 (3.6)	2.4 (5.9)
		Small intestinal		5.2 (2.2)	5 (2.2)	7 (2.7)
		Combined		5.1 (2.3)	4.9 (2)	7 (2.5)
	Non-strangulating	Large intestinal		6.2 (1.6)	6.2 (0.9)	8.3 (1.3)
		Small intestinal		4.3 (1.2)	4.6 (1.1)	8.7 (2)
		Combined		5.6 (1.7)	5.7 (1.2)	8.4 (1.3)
Heart Rate (bpm)	Strangulating	Large intestinal	50 (5)	42 (5)	37 (2)	37 (4)
		Small intestinal	63 (18)	48 (22)	43 (9)	40 (8)
		Combined	60 (17)	47 (20)	41 (8)	39 (7)
	Non-strangulating	Large intestinal	59 (24)	45 (9)	38 (7)	40 (5)
		Small intestinal	46 (10)	39(5)	39 (4)	44 (6)
		Combined	56 (22)	43 (8)	38 (6)	41 (6)

All values are reported as mean +/− standard deviation. Values are reported in the units denoted in parentheses in the Postoperative Variable Column. The four timepoints are denoted as T0, T1, T2, and T3 with their corresponding time post-admission displayed in parentheses.

**Table 2:** Mean +/− standard deviation of peritoneal postoperative variables collected preoperatively and selected times postoperatively in horses undergoing exploratory celiotomy for colic.

Postoperative Variable	Subclassification		T0 (Admission—0 h)	T1 (48 h)	T2 (72 h)
Lactate (mmol/L)	Strangulating	Large intestinal	2.1 (1.3)		
		Small intestinal	5.2 (2.9)	3 (2.1)	2.2 (1.5)
		Combined	4.8 (2.9)	2.8 (2)	2.3 (1.3)
	Non-strangulating	Large intestinal	2.5 (1.2)	2.6 (1.5)	1.4 (0.1)
		Small intestinal	3.4 (0.9)		
		Combined	2.7 (1.1)	2.8 (1.4)	1.4 (0.1)
Total Protein (g/dL)	Strangulating	Large intestinal	1.9 (1.3)		
		Small intestinal	3.4 (1.4)	3 (1)	3.4 (0.6)
		Combined	3.2 (1.4)	3 (1)	3.4 (0.6)
	Non-strangulating	Large intestinal	2.2 (1)	3.8 (0.6)	5.4 (1)
		Small intestinal	1.9 (0.9)		
		Combined	2 (1)	4 (0)	5 (1)
Glucose (mg/dL)	Strangulating	Large intestinal			
		Small intestinal		101 (43)	
		Combined		101 (43)	126 (59)
	Non-strangulating	Large intestinal		84 (33)	102 (15)
		Small intestinal			
		Combined		93 (35)	102 (15)
Total Nucleated Cell Count (/uL)	Strangulating	Large intestinal			
		Small intestinal		23,567 (36,559)	14,140 (2758)
		Combined		23,567 (36,559)	9607 (8.090)
	Non-strangulating	Large intestinal		13,570 (5926)	
		Small intestinal			
		Combined		14,350 (4402)	
Peritoneal: Venous Blood Lactate Ratio	Strangulating	Large intestinal	1.6 (0.4)		
		Small intestinal	2.5 (1)	2.2 (0.9)	2.4 (1.3)
		Combined	2.3 (1)	2.2 (0.9)	2.3 (1.2)
	Non-strangulating	Large intestinal	1.6 (0.7)	2.8 (1.4)	1.5 (0.5)
		Small intestinal	2.1 (0.9)		
		Combined	1.6 (0.7)	2.8 (1.4)	1.5 (0.5)
Peritoneal—Venous Blood Lactate Difference (mmol/L)	Strangulating	Large intestinal	0.9 (0.8)		
		Small intestinal	2.7 (1.7)	1.7 (1.3)	1.3 (1.3)
		Combined	2.5 (1.8)	1.5 (1.3)	1.3 91.1)
	Non-strangulating	Large intestinal	0.8 (0.6)	1.6 (1.4)	0.4 (0.3)
		Small intestinal	1.5 (0.8)		
		Combined	1 (0.7)	1.7 (1.2)	0.4 (0.3)
Peritoneal—Venous Blood Glucose Difference (mg/dL)	Strangulating	Large intestinal			
		Small intestinal		31 (28)	22 (23)
		Combined		31 (28)	30 (0)
	Non-strangulating	Large intestinal		17 (16)	19 (10)
		Small intestinal			
		Combined		22 (18)	19 (10)
Gross Peritoneal Fluid Appearance	Strangulating	Large intestinal	Serosanguinous	Serosanguinous	Serosanguinous
		Small intestinal	Serosanguinous	Serosanguinous	Serosanguinous
		Combined	Serosanguinous	Serosanguinous	Serosanguinous
	Non-strangulating	Large intestinal	Variable	Serosanguinous	Serosanguinous
		Small intestinal	Variable	Serosanguinous	Serosanguinous
		Combined	Variable	Serosanguinous	Serosanguinous

All values are reported as mean +/− standard deviation. Values are reported in the units denoted in parentheses in the Postoperative Variable Column. The three timepoints are denoted as T0, T1, and T2 with their corresponding time post-admission displayed in parentheses.

**Table 3:** Mean (+/− standard deviation) of selected ultrasonographic variables collected preoperatively and selected times postoperatively in horses undergoing exploratory celiotomy for colic.

Small Intestinal Measurement	Subclassification		T0 (Admission—0 h)	T1 (48 h)	T2 (72 h)	T3 (120 h)
Lumen Diameter (mm)	Strangulating	Large intestinal	3.6 (1.4)	2.3 (0.5)	2.8 (1)	2.3 (0.3)
		Small intestinal	6 (1.1)	4.5 (1.6)	4.1 (1.4)	2.8 (0.5)
		Combined	5.4 (1.5)	3.9 (1.7)	3.8 (1.4)	2.7 (0.5)
	Non-strangulating	Large intestinal	3.2 (1.4)	3.4 (0.9)	3.5 (1.3)	2.7 (0.5)
		Small intestinal	5.2 (1.6)	4.8 (0.7)	4 (1.3)	3.5 (0.8)
		Combined	3.7 (1.6)	3.7 (1)	3.6 (1.2)	2.8 (0.6)
Mural Thickness (mm)	Strangulating	Large intestinal	2 (0)	2 (0)	3 (1.7)	2 (0)
		Small intestinal	5.6 (5.8)	3.6 (2.2)	3.2 (1.9)	2.6 (1.2)
		Combined	4.8 (5.3)	3.3 (2)	3.1 (1.8)	2.5 (1)
	Non-strangulating	Large intestinal	2.6 (1.2)	2.6 (1.3)	2.6 (1)	2.3 (0.9)
		Small intestinal	3.3 (1.5)	3 (2)	2 (0)	3.5 (2)
		Combined	2.8 (1.2)	2.7 (1.4)	2.5 (0.8)	2.5 (1.1)

All values are reported as mean +/− standard deviation. Values are reported in the units denoted in parentheses in the Small Intestinal Measurement Column. The four timepoints are denoted as T0, T1, T2, and T3 with their corresponding time post-admission displayed in parentheses.

**Table 4:** Distribution and severity of complications across different intestinal lesion types.

Gastrointestinal Lesion	Classification	None (0)	Mild (1)	Moderate (2)	Severe (3)	Death (4)
Strangulating	Large Intestinal	1/36	3/36	0/36	0/36	0/36
	Small Intestinal	4/36	3/36	3/36	1/36	2/36
	Combined	5/36	6/36	3/36	1/36	2/36
Non-Strangulating	Large Intestinal	3/36	5/36	4/36	1/36	1/36
	Small Intestinal	0/36	4/36	0/36	0/36	1/36
	Combined	3/36	9/36	4/36	1/36	2/36

All values are reported as n/N (where n = #cases that met criteria and N = total #cases). Complications were categorized using a modified accordion severity classification system: no complications (score of 0), mild complications (score of 1), moderate complications (score of 2), severe complications (score of 3), and death (score of 4).

## Data Availability

The original contributions presented in this study are included in the article. Further inquiries can be directed to the corresponding authors.

## Supplementary Materials

The following supporting information can be downloaded at: https://www.mdpi.com/article/10.3390/ani16172720/s1, Table S1: Distribution of small intestinal contractility across different intestinal lesion types preoperatively and selected times postoperatively in horses undergoing exploratory celiotomy for colic.

## Abbreviations

The following abbreviations are used in this manuscript:

FLASH	Fast localized abdominal sonography of horses
PF	Peritoneal fluid
SAA	Serum amyloid A
TNCC	Total nucleated cell count
TP	Total protein
SI	Small intestine
