# Supplementary material for: Serial Postoperative Blood and Peritoneal Fluid Analysis and Ultrasonographic Assessment in Client-Owned Horses Undergoing Ventral Midline Exploratory Celiotomy for Colic
Source: Animals (Basel). 2026 Sep 1;16(17):2720. doi: 10.3390/ani16172720 (PMC13565519; doi:10.3390/ani16172720)
Supplement: Supplementary file 1 [file animals-16-02720-s001.zip › animals-4429813-supplementary.pdf]

Table S1.

Distribution of small intestinal contractility across different intestinal lesion types preoperatively and selected times postoperatively in horses undergoing exploratory celiotomy for colic.

**Lesion Type**		**T0 (Admission – 0 hours)**	**T1 (48 hours)**	**T2 (72 hours)**	**T3 (120 hours)**
Strangulating	Large intestinal	A:<br>H: 3/4<br>N: 1/4	A:<br>H: 1/3<br>N: 2/3	A:<br>H: 1/3<br>N: 2/3	A:<br>H:<br>N: 3/3
	Small intestinal	A: 12/14<br>H: 2/14<br>N:	A: 2/13<br>H: 6/13<br>N: 5/13	A: 1/13<br>H: 6/13<br>N: 6/13	A:<br>H: 4/12<br>N: 8/12
	Combined	A: 12/18<br>H: 5/18<br>N: 1/18	A: 2/16<br>H: 7/16<br>N: 7/16	A: 1/16<br>H: 7/16<br>N: 1/16	A:<br>H: 4/15<br>N: 11/15
Non-strangulating	Large intestinal	A: 1/14<br>H: 4/14<br>N: 9/14	A:<br>H: 9/14<br>N: 5/14	A:<br>H: 4/13<br>N: 9/13	A:<br>H:<br>N: 10/10
	Small intestinal	A: 2/4<br>H: 1/4<br>N: 1/4	A: 1/4<br>H: 3/4<br>N:	A: 1/4<br>H: 2/4<br>N: 1/4	A:<br>H:<br>N: 2/2
	Combined	A: 3/18<br>H: 5/18<br>N: 10/18	A: 1/18<br>H: 12/18<br>N: 5/18	A: 1/17<br>H: 6/17<br>N: 10/17	A:<br>H:<br>N: 12/12

All values are reported as n/N (where n = # ultrasonographic examinations that met criteria and N = total #ultrasonographic examinations available). A= absent, H= hypomotile, and N= normal. The four time points are denoted as T0, T1, T2, T3 with their corresponding time post admission displayed in parentheses.
